# Prognostic Significance of Dynamic CZT Cardiac-Dedicated SPECT-derived Myocardial Flow Reserve in Patients with Suspected or Confirmed Coronary Artery Disease and Normal Myocardial Perfusion

**DOI:** 10.2174/0115734056423358251204111943

**Published:** 2026-01-13

**Authors:** Yingfei Liu, Fukai Zhao, Zekun Pang, Yue Chen, Jiao Wang, Jianming Li

**Affiliations:** 1 Department of Nuclear Medicine, TEDA International Cardiovascular Hospital, Tianjin Medical University Cardiovascular Institute, Tianjin, China

**Keywords:** Cadmium-zinc-telluride, Single photon emission computed tomography, Myocardial flow reserve, Coronary artery disease, OCAD, CZT

## Abstract

**Introduction:**

This study aimed to evaluate the prognostic significance of dynamic cadmium-zinc-telluride (CZT) cardiac-dedicated single photon emission computed tomography (SPECT)-derived myocardial flow reserve (MFR) in patients with suspected or confirmed coronary artery disease (CAD) and normal myocardial perfusion.

**Methods:**

A consecutive cohort of patients who completed dynamic myocardial perfusion imaging and routine myocardial perfusion imaging (MPI) using CZT cardiac-dedicated SPECT were selected and followed up for at least 24 months to determine the occurrence of major adverse cardiac events (MACEs). Patients were divided into groups with no MACEs and MACEs. Differences between the two groups in baseline characteristics, semiquantitative, and quantitative parameters were compared. Cox regression analysis was performed to identify predictive factors associated with MACEs. Kaplan-Meier survival curves were plotted, and log-rank tests were performed to compare the incidence of MACEs between the normal MFR group and the reduced MFR group.

**Results:**

A total of 369 patients with negative routine MPI results were included in this study, with an average age of 61.82±8.68 years (113 males and 256 females). The median follow-up duration was 30 months [IQR (25, 34)], during which 73 patients experienced MACEs. The incidence of MACEs was significantly higher in patients with reduced MFR than in those with normal MFR (*P*<0.05). Cox regression analysis identified reduced MFR as an independent predictor of MACEs (HR: 2.076, 95%CI: 1.174-3.669, *P*=0.012). The proportion of patients diagnosed with obstructive coronary artery disease (OCAD) was significantly higher in the MACE group compared to the no MACE group (*P*<0.05).

**Discussion:**

These findings provide critical clinical insights, particularly for patients in whom myocardial ischemia is not detected *via* traditional semiquantitative MPI analysis. CZT cardiac-dedicated SPECT, which enables quantitative assessment of myocardial blood flow, serves as a more precise tool for early CAD diagnosis and prognostic evaluation. This underscores the role of CZT cardiac-dedicated SPECT in assessing myocardial ischemia and prognosis among patients with negative conventional MPI, helping to identify high-risk individuals that conventional MPI may overlook. By leveraging CZT cardiac-dedicated SPECT to obtain absolute quantitative myocardial blood flow (MBF) and MFR, myocardial perfusion is quantified more accurately, thereby overcoming the limitations of traditional MPI and providing a more reliable basis for early clinical diagnosis and treatment.

**Conclusion:**

MFR measured with CZT cardiac-dedicated SPECT can effectively predict the prognosis of patients with suspected or confirmed CAD and normal MPI. Reduced MFR is significantly associated with a higher incidence of MACEs, and MFR reduction is an independent predictor of MACEs.

## 1 INTRODUCTION

Coronary artery disease (CAD) ranks among the most prevalent cardiovascular conditions globally, with its incidence and prevalence steadily increasing and posing a significant threat to human health [1, 2]. Early detection and accurate assessment of the extent and severity of myocardial ischemia are critical for guiding therapeutic decisions and prognostic evaluation in patients with CAD [3]. Noninvasive cardiac imaging modalities, particularly radionuclide myocardial perfusion imaging (MPI), have been increasingly recognized for their pivotal role in CAD diagnosis and therapeutic decision-making [4].

Traditional single-photon emission computed tomography (SPECT) with sodium iodide crystals, a mainstay of MPI, exhibits inherent limitations. This modality primarily relies on semiquantitative analysis of tomographic parameters for diagnosis, largely due to suboptimal image acquisition resolution. Moreover, conventional SPECT is unable to fully and quantitatively evaluate coronary hemodynamics, especially in the context of three-vessel disease assessment [5]. While conventional SPECT MPI effectively detects myocardial ischemia, it carries a risk of underdiagnosing obstructive coronary artery disease (OCAD) in patients with normal myocardial perfusion images [6]. Notably, SPECT MPI interpretation may yield false-negative results in cases of severe stenosis with balanced involvement across all three coronary vessels, potentially leading to a missed diagnosis of “balanced ischemia” [7].

Quantification of myocardial blood flow (MBF) and myocardial flow reserve (MFR) has long been regarded as the unique strength of positron emission tomography (PET) MPI. However, the widespread clinical adoption of PET remains restricted by high equipment and operational costs, limited tracer availability, and relatively high radiation burden. Consequently, there is a strong clinical impetus to explore alternative imaging strategies that can provide PET-like quantitative information but with broader accessibility.

Over the past decade, cardiac-dedicated SPECT systems equipped with solid-state cadmium-zinc-telluride (CZT) semiconductor detectors have been increasingly integrated into clinical practice [4]. CZT cardiac SPECT is characterized by high sensitivity, superior spatial resolution, and lower administered radiotracer doses compared with conventional SPECT. Importantly, its substantially improved temporal resolution enables dynamic acquisitions, thereby facilitating the quantification of absolute MBF and MFR in a manner analogous to PET. Previous studies have demonstrated that CZT SPECT-derived flow quantification shows good agreement with PET while offering practical advantages, such as wider availability and lower cost [8]. This advantage enhances its utility for detecting left main coronary artery disease, multivessel disease, and balanced triple-vessel lesions, thereby mitigating underestimation or underdiagnosis of such OCAD cases [9].

Despite these advancements, evidence supporting the use of cardiac-dedicated CZT SPECT in this specific context remains limited. The present study utilized dynamic CZT cardiac-dedicated SPECT imaging to assess MFR in patients with suspected or confirmed CAD and normal myocardial perfusion. Our aim was to evaluate the prognostic value of MFR in this population and provide an objective basis for developing personalized clinical treatment strategies.

## 2 METHODS

### 2.1 Study Population

This is a retrospective, single-center cohort study. Data were retrospectively collected from consecutively screened patients with suspected or confirmed coronary artery disease (CAD) who underwent cardiac-dedicated SPECT and routine gated myocardial perfusion imaging (MPI) at TEDA International Cardiovascular Hospital between January 2019 and April 2022. A total of 3,127 patients were initially screened, of whom 2,758 were excluded for not meeting the inclusion criteria. Finally, 501 patients were initially enrolled for follow-up; after excluding patients lost to follow-up or who underwent early revascularization, 369 patients (73.7%) were successfully followed and included in the analysis. All patients had negative routine MPI results, and each patient was followed up for at least 24 months to collect follow-up data. Inclusion criteria were as follows: (1) suspected or confirmed CAD; (2) successful completion of dynamic and routine electrocardiogram (ECG)-gated MPI with good image quality; (3) negative MPI test results (summed stress score [SSS] <4; summed difference score [SDS] <2) [10-13]; (4) coronary angiography (CAG) performed within 3 months before or after SPECT imaging without prior or subsequent revascularization. Exclusion criteria were: (1) absence of CAG data within 3 months before or after the examination; (2) history of old myocardial infarction; (3) prior coronary artery bypass grafting; (4) suspected or confirmed cardiomyopathy, valvular heart disease, myocarditis, or coronary microvascular disease (CMD); (5) failure of dynamic image acquisition or quality control; (6) positive MPI test (SSS≥4 or SDS≥2); (7) early revascularization (percutaneous coronary intervention or coronary artery bypass grafting) within 3 months of the examination; (8) loss of clinical data. Demographic and clinical characteristics of enrolled patients were collected, including hypertension, hyperlipidemia, diabetes, smoking history, body mass index (BMI), family history of CAD, and clinical symptoms. Image data included MPI parameters, quantitative flow parameters, and CAG findings. This retrospective study was conducted with approval from the Institutional Ethics Committee of TEDA International Cardiovascular Hospital (No. 2025-0526-1) and with proper informed consent from all participants. The research strictly adhered to the principles outlined in the Declaration of Helsinki.

### 2.2 SPECT Imaging

Prior to SPECT imaging, patients were instructed to avoid coffee, tea, and any foods containing caffeine or theophylline and to discontinue cardiovascular medications for 24 hours. Dynamic SPECT imaging and MPI were performed using a GE NM530C SPECT camera (GE Healthcare, Haifa, Israel) with 99mTc-MIBI tracer. One-day or two-day imaging protocols combining dynamic SPECT with MPI were employed, as previously described in other studies [14, 15]. Pharmacologic vasodilator stress was induced using adenosine triphosphate (ATP, 0.16 mg/kg/min for 5 minutes) or adenosine (0.14 mg/kg/min for 6 minutes). During the peak of pharmacologic stress (3 minutes after infusion start), the stress tracer dose was approximately 3–4 times the rest dose. List-mode data from dynamic SPECT and low-dose CT (120 kV, 20 mA; GE NM690, GE Healthcare, Milwaukee, WI, USA) for attenuation correction were transferred to the MyoFlowQ workstation (Beijing Lark Cloud Biomedicals, Beijing, China) for image reconstruction and myocardial blood flow (MBF) quantification. Dynamic data were reconstructed into a series of frames (10 frames/10 s, 5 frames/20 s, 2 frames/60 s, 1 frame/280 s), with fusion of CT-based attenuation correction. Regions of interest (ROIs) were defined for the left ventricular cavity to generate the input function and for myocardial walls at the basal segments. MBF was calculated for the entire left ventricle and for each coronary artery territory (LAD, LCX, RCA) under rest (rMBF) and stress (sMBF) conditions, in units of mL/g/min. Myocardial flow reserve (MFR) was defined as sMBF/rMBF, and relative flow reserve (RFR) was calculated for each coronary territory. Two experienced nuclear cardiologists, blinded to patients’ clinical information and coronary angiography (CAG) results, independently reviewed the SPECT images. Discrepancies were resolved by consensus.

Conventional gated MPI images were processed using an iterative reconstruction method. Quantitative perfusion SPECT (QPS) and quantitative gated SPECT (QGS) software (Cedars Sinai Medical Center, CA, LA, USA) were used to generate left ventricular (LV) horizontal long-axis, vertical long-axis, and short-axis images, as well as 17-segment target maps according to American Heart Association (AHA) standards. Radioactivity distribution in each myocardial segment of the target heart maps was assessed using a standard 5-point scale: 0=normal, 1=mild, 2=moderate, 3=severe reduction of tracer uptake, and 4=absent tracer uptake. Semiquantitative MPI parameters (SSS, summed rest score [SRS], SDS, and transient ischemic dilatation [TID]) were analyzed *via* QPS. LV functional parameters (end-diastolic volume [EDV], end-systolic volume [ESV], left ventricular ejection fraction [LVEF], peak ejection rate [PER], and peak filling rate [PFR]) were calculated from QGS at both stress and rest.

### 2.3 CAG

In the clinical setting, two experienced interventional cardiologists analyzed CAG results and finalized the angiographic report. Patients were categorized into two groups based on CAG findings: obstructive CAD (OCAD), defined as left main coronary artery stenosis ≥50% or ≥70% in any of the three coronary arteries; and non-OCAD (NOCAD), otherwise. The interventional cardiologists who analyzed CAG results were blinded to the SPECT findings.

### 2.4 Patients' Follow-up

Follow-up data were collected using both hospital medical records and telephone interviews. Cardiovascular deaths were adjudicated based on hospital records, medical documentation, and death certificates when available. The median follow-up duration after CZT cardiac-specific SPECT imaging was 30 months (interquartile range [IQR]: 25–34). Major adverse cardiac events (MACEs) were defined as the occurrence of at least one of the following: cardiovascular death, nonfatal myocardial infarction, first-onset heart failure, rehospitalization for angina pectoris, or advanced coronary revascularization (percutaneous coronary intervention or coronary artery bypass grafting) [16-18]. Myocardial infarction was defined by meeting at least two criteria: chest pain or equivalent symptoms, positive cardiac biomarkers, or typical electrocar-diographic changes [19]. Follow-up duration was determined using the date of the last examination or consultation.

### 2.5 Statistical Analysis

Patients were divided into MACE and non-MACE groups based on follow-up outcomes. Additionally, patients were stratified into normal MFR (MFR ≥2.0) and reduced MFR (MFR <2.0) groups according to global LV MFR [20]. As this study is a retrospective investigation, no formal sample size or power calculation was performed; the sample size was determined by the number of patients meeting inclusion and exclusion criteria during the study period. Continuous variables were tested for normality using the Shapiro-Wilk test; all continuous variables were normally distributed and presented as mean±standard deviation (SD), and categorical variables were presented as frequency and percentage. Categorical variables were compared using the χ^2^ test, and continuous variables using the independent samples t-test. Cumulative MACE incidence was estimated *via* the Kaplan-Meier method and compared using the log-rank test, with time from inclusion to MACE onset as the analysis endpoint. All variables were first evaluated *via* univariate Cox proportional hazards regression. Baseline variables deemed clinically relevant or showing univariate associations with outcomes were included in the multivariate Cox proportional hazards regression model. Results were reported as hazard ratio (HR) with 95% confidence interval (95% CI). Statistical significance was defined as *P*<0.05. All analyses were performed using SPSS (version 27.0.1, SPSS Inc., Chicago, IL, USA).

## 3 RESULTS

### 3.1 Patient Characteristics

Our study included 369 patients with negative routine MPI results. Among these, 113 (30.6%) were male, and 256 (69.4%) were female, with ages ranging from 33 to 82 years (mean±SD: 61.82±8.68). MACEs occurred in 73 patients (73/369, 19.8%), including 1 (1.4%) cardiovascular death, 1 (1.4%) nonfatal myocardial infarction, 1 (1.4%) heart failure, 48 (65.8%) rehospitalizations for angina pectoris, and 22 (30.1%) advanced coronary revascularization. The overall incidence of obstructive coronary artery disease (OCAD) in the cohort was 33.3% (123/369). The proportion of OCAD was significantly higher in the MACE group than in the non-MACE group (43.8% *vs*. 30.7%, *P*<0.05). No significant differences were observed between the two groups in baseline characteristics, clinical symptoms, or cardiovascular risk factors (all P>0.05) (Table **1**).

### 3.2 Semiquantitative Perfusion and Quantitative Flow Parameters

Comparisons of semiquantitative and quantitative parameters between the two groups are presented in Table **2**. The proportion of patients with reduced myocardial flow reserve (MFR) was significantly higher in the MACE group than in the non-MACE group (20.5% *vs*. 10.1%, *P*<0.05). No significant differences were observed in the remaining semiquantitative or quantitative parameters between the two groups (all P>0.05).

### 3.3 Prognostic Value of MFR

Within the study population, the incidence of MACEs was significantly higher among patients with reduced MFR than among those with normal MFR (33.3% *vs*. 17.9%, *P*=0.015). Univariate Cox regression analysis of the parameters listed above identified males (HR: 1.616; 95% CI: 1.010–2.583; *P*=0.045), OCAD (HR: 1.737; 95% CI: 1.093–2.759; *P*=0.019), and reduced MFR (HR: 2.076; 95% CI: 1.174–3.669; *P*=0.012) as significant predictors of MACEs. Multivariate Cox regression analysis, which included males, reduced MFR, and OCAD, confirmed that reduced MFR (HR: 2.076; 95% CI: 1.174–3.669; *P*=0.012) was an independent predictor of MACEs. Results of the Cox regression analyses are presented in Table **3**. Kaplan-Meier survival analysis revealed that patients with reduced MFR (log-rank=6.654, *P*=0.010) had the highest risk of MACEs (Fig. **1**).

## 4 DISCUSSION

This study evaluated the predictive significance of MFR measured by CZT cardiac-dedicated SPECT for MACEs in patients with suspected or confirmed CAD and negative MPI. Our results confirmed a significantly higher incidence of MACEs in patients with reduced MFR compared to those with normal MFR. Multivariate Cox regression analysis further identified reduced MFR as an independent predictor of MACEs (HR: 2.076; 95% CI: 1.174–3.669; *P*=0.012), indicating that patients with reduced MFR have a 2.076-fold greater risk of MACEs than those with normal MFR, corresponding to a 107.6% increase in risk. Importantly, these findings demonstrate that patients with negative conventional MPI but reduced MFR cannot be considered low-risk, as they exhibit a substantially increased risk of adverse events. Therefore, while routine invasive coronary angiography may not be necessary for all such patients, closer clinical surveillance or selective invasive assessment could be considered, particularly for patients with persistent symptoms or additional high-risk features. Although there were no statistically significant differences in mean sMBF and MFR values between the MACE and non-MACE groups in the baseline comparison, survival analysis using Kaplan–Meier curves and Cox regression demonstrated that patients with reduced MFR (<2.0) had a significantly higher risk of MACEs during follow-up. This indicates that while cross-sectional group differences may not reach statistical significance due to sample size and variability, stratification by the clinically established MFR threshold (2.0) revealed its prognostic value. Additionally, we observed a significantly higher proportion of OCAD in the MACE group versus the non-MACE group, suggesting that even in patients with normal MPI results, CZT cardiac-dedicated SPECT-derived MFR effectively identifies potentially high-risk individuals [21, 22].

These findings provide critical clinical insights, particularly for patients in whom myocardial ischemia is not detected *via* traditional semiquantitative MPI analysis. CZT cardiac-dedicated SPECT, which enables quantitative assessment of myocardial blood flow, serves as a more precise tool for early CAD diagnosis and prognostic evaluation [23]. This underscores the role of CZT cardiac-dedicated SPECT in assessing myocardial ischemia and prognosis among patients with negative conventional MPI, helping to identify high-risk individuals that conventional MPI may overlook [24, 25]. By leveraging CZT cardiac-dedicated SPECT to obtain absolute quantitative myocardial blood flow (MBF) and MFR, myocardial perfusion is quantified more accurately, thereby overcoming the limitations of traditional MPI and providing a more reliable basis for early clinical diagnosis and treatment [26].

Souza *et al*. [7] used CZT cardiac-dedicated SPECT to quantify coronary flow reserve, demonstrating its efficacy in evaluating multivessel coronary artery lesions. Their work highlighted that CZT technology provides quantitative insights into coronary hemodynamics, facilitating more accurate condition assessment. Similarly, Wang *et al*. [9] emphasized the advantages of CZT cardiac-dedicated SPECT in quantifying MBF and MFR, noting that CZT dynamic imaging is more efficient than conventional semiquantitative assessment. Pang *et al*. [14] explored the diagnostic value of low-dose dynamic myocardial perfusion imaging using CZT cardiac-dedicated SPECT for detecting novel quantitative parameters in patients with suspected or known CAD. They found that CZT cardiac-dedicated SPECT yields comprehensive quantitative data, including MBF and MFR, which are instrumental in more precisely evaluating the severity and extent of myocardial ischemia. Although these studies did not directly address prognostic assessment in OCAD patients with negative conventional MPI, their results strongly support the use of CZT cardiac-dedicated SPECT for the evaluation of myocardial ischemia.

## 5 LIMITATIONS

This study has several limitations. First, it is a retrospective single-center investigation with a relatively small sample size, which may introduce selection and information bias. Second, while CZT cardiac-dedicated SPECT offers the advantage of absolute myocardial blood flow quantification, this study did not include comparative analyses with other emerging imaging techniques. Moreover, the absence of quantitative coronary angiography (QCA) and fractional flow reserve (FFR), the gold-standard techniques for assessing coronary stenosis severity and hemodynamic significance, limits the ability to evaluate the true anatomical and functional impact of coronary lesions. In addition, inter- and intra-observer reproducibility for MBF/MFR quantification was not evaluated in this study, which should be addressed in future research. Finally, because the study population was limited to patients with negative conventional MPI, the generalizability of our findings to broader CAD cohorts may be restricted. Consequently, the specific clinical advantages of CZT technology require further clarification in future studies.

## CONCLUSION

In summary, this study demonstrates that in patients with OCAD and negative routine MPI (*via* semiquantitative analysis), quantitatively determined MFR reduction is significantly associated with MACE occurrence, and reduced MFR serves as an independent predictor of MACEs. Thus, absolute myocardial flow quantification by CZT cardiac-dedicated SPECT enables a more comprehensive assessment of cardiovascular function and effectively evaluates myocardial flow reserve in patients with suspected or confirmed CAD and negative routine MPI (semiquantitative analysis). This provides a simple, quantitative, noninvasive imaging tool for accurate diagnosis and prognostic assessment in such patients.

## Figures and Tables

**Fig. (1):**
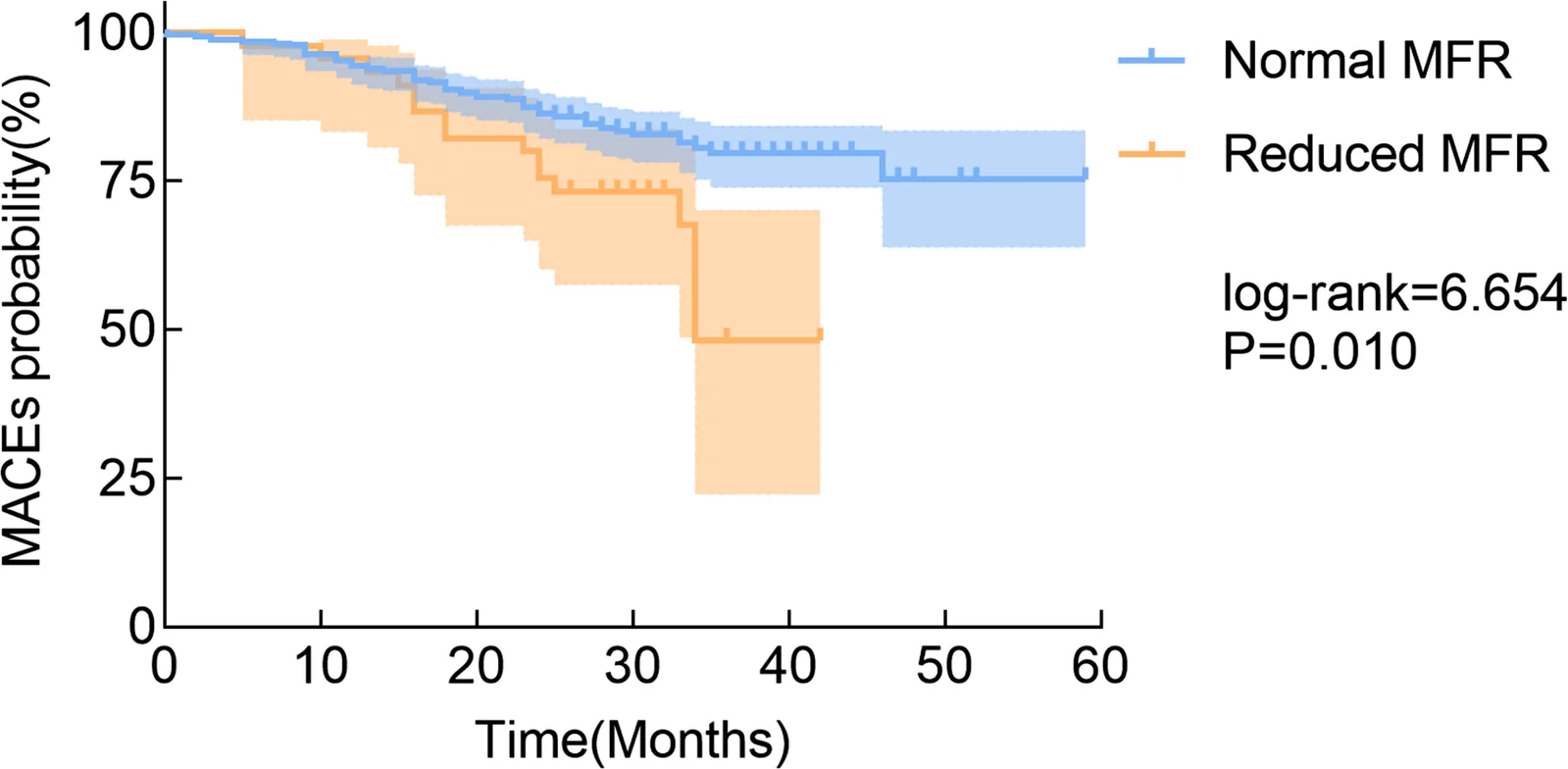
Kaplan-Meier survival analysis between the normal MFR and reduced MFR groups.

**Table 1:** Comparisons of patients' characteristics between non-MACE and MACE groups.

	**Non-MACE Groups** **(n=296)**	**MACE Groups** **(n=73)**	***P* Value**
Demographics			
Age, (years)	61.63±8.86	62.63±7.94	0.376
Male, n (%)	84(28.4%)	29(39.7%)	0.060
BMI (kg/m^2^)	25.82±3.44	25.50±3.32	0.483
Clinical symptoms			0.404
Typical angina pectoris, n (%)	31(10.5%)	11(15.1%)	
Atypical angina pectoris, n (%)	250(84.5%)	60(82.2%)	
No angina pectoris, n (%)	15(5.1%)	2(2.7%)	
Risk factors			
Hypertension, n (%)	179(60.5%)	48(65.8%)	0.406
Dyslipidemia, n (%)	131(44.3%)	41(56.2%)	0.068
Diabetes, n (%)	69(23.3%)	23(31.5%)	0.147
Smoking, n (%)	73(24.7%)	23(31.5%)	0.233
Family history, n (%)	97(32.8%)	26(35.6%)	0.644

**Note**: *P<0.05

**Table 2:** Comparisons of semi-quantitative and quantitative parameters between non-MACE and MACE groups.

	**Non-MACE Group** **(n=296)**	**MACE Groups** **(n=73)**	***P* Value**
**Semi-quantitative parameters**			
sEDV (mL)	71.76±20.12	70.97±19.62	0.763
sESV (mL)	25.95±12.50	25.21±11.80	0.644
sLVEF (%)	65.31±8.48	65.99±8.68	0.547
sPER (-EDV/s)	-3.43±0.67	-3.48±0.70	0.523
sPFR (EDV/s)	2.79±0.77	2.67±0.82	0.209
rEDV (mL)	66.38±19.28	66.29±18.21	0.972
rESV (mL)	23.74±11.72	23.22±10.99	0.733
rLVEF (%)	65.72±8.60	66.58±8.54	0.446
rPER (-EDV/s)	-3.25±0.65	-3.37±0.74	0.173
rPFR (EDV/s)	2.50±068	2.46±0.64	0.652
TID	1.15±0.14	1.15±0.14	0.829
**Quantitative parameters**			
rMBF (mL/min/g)	0.89±0.11	0.89±0.15	0.945
sMBF (mL/min/g)	2.56±0.78	2.43±0.83	0.202
MFR	2.85±0.78	2.72±0.86	0.189
Reduced MFR, n (%)	30(10.1%)	15(20.5%)	0.015*

**Note:** *P<0.05.

**Table 3:** Cox regression analysis of different parameters predicting MACEs in all patients (n=369).

**Variables**	**Univariate Cox**		**Multivariate Cox**	
	**HR (95%CI)**	***P* Value**	**HR (95%CI)**	***P* Value**
Age (years)	1.011(0.985-1.039)	0.411		
Male (%)	1.616(1.010-2.583)	0.045*		
BMI (kg/m^2^)	0.977(0.913-1.045)	0.492		
Hypertension (%)	1.211(0.747-1.964)	0.438		
Dyslipidemia (%)	1.516(0.955-2.408)	0.078		
Diabetes (%)	1.424(0.869-2.333)	0.161		
Smoking (%)	1.360(0.830-2.229)	0.222		
Family history (%)	1.148(0.711-1.854)	0.573		
OCAD (%)	1.737(1.093-2.759)	0.019*		
sEDV (mL)	0.998(0.986-1.010)	0.713		
sESV (mL)	0.996(0.977-1.015)	0.667		
sLVEF (%)	1.007(0.980-1.035)	0.662		
sPER (-EDV/s)	0.837(0.598-1.171)	0.299		
sPFR (EDV/s)	0.862(0.628-1.184)	0.359		
rEDV (mL)	0.999(0.987-1.011)	0.882		
rESV (mL)	0.997(0.976-1.017)	0.750		
rLVEF (%)	1.009(0.982-1.037)	0.526		
rPER (-EDV/s)	0.777(0.549-1.099)	0.153		
rPFR (EDV/s)	0.924(0.651-1.310)	0.657		
TID	0.889(0.180-4.399)	0.885		
rMBF (mL/min/g)	0.986(0.134-7.237)	0.989		
sMBF (mL/min/g)	0.828(0.614-1.116)	0.215		
MFR	0.822(0.610-1.110)	0.201		
Reduced MFR	2.076(1.174-3.669)	0.012*	2.076(1.174-3.669)	0.012*

**Note:** *P<0.05.

## Data Availability

All the data and supporting information are provided within the article.

## LIST OF ABBREVIATIONS

CAD: Coronary artery disease
MPI: Myocardial perfusion imaging
SPECT: Single photon emission computed tomography
OCAD: Obstructive coronary artery disease
NOCAD: Non-obstructive coronary artery disease
CZT: Cadmium-zinc-telluride
PET: Positron emission tomography
MBF: Myocardial blood flow
MFR: Myocardial flow reserve
SSS: Summed stress scores
SDS: Summed difference scores
SRS: Summed rest scores
CAG: Coronary angiography
CMD: Coronary microvascular disease
BMI: Body mass index
CT: Computed tomography
QPS: Quantitative perfusion SPECT
QGS: Quantitative gated SPECT
AHA: American Heart Association
TID: Transient ischemic dilatation
LV: Left ventricular
EDV: End-diastolic volume
ESV: End-systolic volume
LVEF: Left ventricular ejection fraction
PER: Peak ejection rate
PFR: Peak filling rate
MACEs: Major adverse cardiac events
SD: Standard deviation
HR: Hazard ratio
95% CI: 95% confidence interval

## STANDARDS OF REPORTING

STROBE guidelines were followed.

## References

[1] Houssany-Pissot S., Rosencher J., Allouch P., Bensouda C., Pillière R., Cacoub L., Caussin C., El-Hadad S., Makowski S., Beverelli F., Cacoub P. (2020). Screening coronary artery disease with computed tomography angiogram should limit normal invasive coronary angiogram, regardless of pretest probability.. Am. Heart J..

[2] Wang J., Yang Z.G., Guo Y.K., Jiang Y., Yan W.F., Qian W.L., Fang H., Min C.Y., Li Y. (2023). Incremental effect of coronary obstruction on myocardial microvascular dysfunction in type 2 diabetes mellitus patients evaluated by first-pass perfusion CMR study.. Cardiovasc. Diabetol..

[3] Ties D., van Dorp P., Pundziute G., van der Aalst C.M., Gratama J.W.C., Braam R.L., Kuijpers D., Lubbers D.D., van der Bilt I.A.C., Westenbrink B.D., Wolcherink M.J.O., Doggen C.J.M., Išgum I., Nijveldt R., de Koning H.J., Vliegenthart R., Oudkerk M., van der Harst P. (2022). Early detection of obstructive coronary artery disease in the asymptomatic high-risk population: Objectives and study design of the EARLY-SYNERGY trial.. Am. Heart J..

[4] Konsek-Komorowska S.J., Peczkowska M., Cwikła J.B. (2022). Myocardial perfusion imaging using single-photon emission computed tomography with cadmium-zinc-telluride technology.. Nuclear Medicine Review.

[5] Hung G-U., Wang Y-F., Su H-Y., Hsieh T-C., Ko C-L., Yen R-F. (2016). New trends in radionuclide myocardial perfusion imaging.. Zhonghua Minguo Xinzangxue Hui Zazhi.

[6] Hung G.U., Ko K.Y., Lin C.L., Yen R.F., Kao C.H. (2018). Impact of initial myocardial perfusion imaging versus invasive coronary angiography on outcomes in coronary artery disease: A nationwide cohort study.. Eur. J. Nucl. Med. Mol. Imaging.

[7] Souza A.C.A.H., Gonçalves B.K.D., Tedeschi A., Lima R.S.L. (2018). Quantification of coronary flow reserve with CZT gamma camera in the evaluation of multivessel coronary disease.. Arq. Bras. Cardiol..

[8] Agostini D., Roule V., Nganoa C., Roth N., Baavour R., Parienti J.J., Beygui F., Manrique A. (2018). First validation of myocardial flow reserve assessed by dynamic ^99m^Tc-sestamibi CZT-SPECT camera: Head to head comparison with ^15^O-water PET and fractional flow reserve in patients with suspected coronary artery disease. The WATERDAY study.. Eur. J. Nucl. Med. Mol. Imaging.

[9] Wang J., Li S., Chen W., Chen Y., Pang Z., Li J. (2021). Diagnostic efficiency of quantification of myocardial blood flow and coronary flow reserve with CZT dynamic SPECT imaging for patients with suspected coronary artery disease: A comparative study with traditional semi-quantitative evaluation.. Cardiovasc. Diagn. Ther..

[10] Dorbala S., Ananthasubramaniam K., Armstrong I.S., Chareonthaitawee P., DePuey E.G., Einstein A.J., Gropler R.J., Holly T.A., Mahmarian J.J., Park M.A., Polk D.M., Russell R., Slomka P.J., Thompson R.C., Wells R.G. (2018). Single photon emission computed tomography (SPECT) myocardial perfusion imaging guidelines: Instrumentation, acquisition, processing, and interpretation.. J. Nucl. Cardiol..

[11] Tilkemeier P.L., Bourque J., Doukky R., Sanghani R., Weinberg R.L. (2017). ASNC imaging guidelines for nuclear cardiology procedures.. J. Nucl. Cardiol..

[12] Kolkailah A.A., Iskander M., Iskander F., Patel P.P., Khan R., Doukky R. (2022). The prognostic utility of regadenoson SPECT myocardial perfusion imaging in patients with end-stage renal disease: The largest cohort to date.. J. Nucl. Cardiol..

[13] Doukky R., Olusanya A., Vashistha R., Saini A., Fughhi I., Mansour K., Nigatu A., Confer K., Sims S.A. (2015). Diagnostic and prognostic significance of ischemic electrocardiographic changes with regadenoson-stress myocardial perfusion imaging.. J. Nucl. Cardiol..

[14] Pang Z., Wang J., Li S., Chen Y., Wang X., Li J. (2021). Diagnostic analysis of new quantitative parameters of low-dose dynamic myocardial perfusion imaging with CZT SPECT in the detection of suspected or known coronary artery disease.. Int. J. Cardiovasc. Imaging.

[15] Wang J., Chen Y., Chu H., Pang Z., Hsu B., Li J. (2024). Feasibility of myocardial blood flow quantification to detect flow-limited coronary artery disease with a one-day rest/stress continuous rapid imaging protocol on cardiac-dedicated cadmium zinc telluride single photon emission computed tomography.. J. Nucl. Cardiol..

[16] Zampella E., Assante R., D’Antonio A., Mannarino T., Gaudieri V., Nappi C., Arumugam P., Panico M., Buongiorno P., Petretta M., Cuocolo A., Acampa W. (2024). Prognostic value of coronary flow capacity by ^82^ Rb PET in patients with suspected coronary artery disease and normal myocardial perfusion at semiquantitative imaging analysis.. Circ. Cardiovasc. Imaging.

[17] Matsuo S., Nakajima K., Takeishi Y., Nishimura T. (2018). Prognostic value of normal stress myocardial perfusion imaging and ventricular function in Japanese patients with chronic kidney disease: A study based on the J-ACCESS-3 database.. Eur. J. Nucl. Med. Mol. Imaging.

[18] Zhang H., Caobelli F., Che W., Huang Y., Zhang Y., Fan X., Hu X., Xu C., Fei M., Zhang J., Lv Z., Shi K., Yu F. (2023). The prognostic value of CZT SPECT myocardial blood flow (MBF) quantification in patients with ischemia and no obstructive coronary artery disease (INOCA): A pilot study.. Eur. J. Nucl. Med. Mol. Imaging.

[19] Thygesen K., Alpert J.S., Jaffe A.S., Chaitman B.R., Bax J.J., Morrow D.A., White H.D., Thygesen K., Alpert J.S., Jaffe A.S., Chaitman B.R., Bax J.J., Morrow D.A., White H.D., Mickley H., Crea F., Van de Werf F., Bucciarelli-Ducci C., Katus H.A., Pinto F.J., Antman E.M., Hamm C.W., De Caterina R., Januzzi J.L., Apple F.S., Alonso Garcia M.A., Underwood S.R., Canty J.M., Lyon A.R., Devereaux P.J., Zamorano J.L., Lindahl B., Weintraub W.S., Newby L.K., Virmani R., Vranckx P., Cutlip D., Gibbons R.J., Smith S.C., Atar D., Luepker R.V., Robertson R.M., Bonow R.O., Steg P.G., O’Gara P.T., Fox K.A.A., Hasdai D., Aboyans V., Achenbach S., Agewall S., Alexander T., Avezum A., Barbato E., Bassand J-P., Bates E., Bittl J.A., Breithardt G., Bueno H., Bugiardini R., Cohen M.G., Dangas G., de Lemos J.A., Delgado V., Filippatos G., Fry E., Granger C.B., Halvorsen S., Hlatky M.A., Ibanez B., James S., Kastrati A., Leclercq C., Mahaffey K.W., Mehta L., Müller C., Patrono C., Piepoli M.F., Piñeiro D., Roffi M., Rubboli A., Sharma S., Simpson I.A., Tendera M., Valgimigli M., van der Wal A.C., Windecker S., Chettibi M., Hayrapetyan H., Roithinger F.X., Aliyev F., Sujayeva V., Claeys M.J., Smajić E., Kala P., Iversen K.K., El Hefny E., Marandi T., Porela P., Antov S., Gilard M., Blankenberg S., Davlouros P., Gudnason T., Alcalai R., Colivicchi F., Elezi S., Baitova G., Zakke I., Gustiene O., Beissel J., Dingli P., Grosu A., Damman P., Juliebø V., Legutko J., Morais J., Tatu-Chitoiu G., Yakovlev A., Zavatta M., Nedeljkovic M., Radsel P., Sionis A., Jemberg T., Müller C., Abid L., Abaci A., Parkhomenko A., Corbett S. (2019). Fourth universal definition of myocardial infarction (2018).. Eur. Heart J..

[20] Ong P., Camici P.G., Beltrame J.F., Crea F., Shimokawa H., Sechtem U., Kaski J.C., Bairey Merz C.N. (2018). International standardization of diagnostic criteria for microvascular angina.. Int. J. Cardiol..

[21] Kassab K., Hussain K., Torres A., Iskander F., Iskander M., Khan R., Doukky R. (2022). The diagnostic and prognostic value of near-normal perfusion or borderline ischemia on stress myocardial perfusion imaging.. J. Nucl. Cardiol..

[22] Acampa W., Assante R., Mannarino T., Zampella E., D’Antonio A., Buongiorno P., Gaudieri V., Nappi C., Giordano A., Mainolfi C.G., Petretta M., Cuocolo A. (2020). Low-dose dynamic myocardial perfusion imaging by CZT-SPECT in the identification of obstructive coronary artery disease.. Eur. J. Nucl. Med. Mol. Imaging.

[23] Cantoni V., Green R., Ricciardi C., Assante R., Donisi L., Zampella E. (2021). Comparing the prognostic value of stress myocardial perfusion imaging by conventional and cadmium-zinc telluride single-photon emission computed tomography through a machine learning approach.. Comput Math Methods Med.

[24] Shiraishi S., Tsuda N., Sakamoto F., Ogasawara K., Tomiguchi S., Tsujita K., Yamashita Y. (2020). Clinical usefulness of quantification of myocardial blood flow and flow reserve using CZT-SPECT for detecting coronary artery disease in patients with normal stress perfusion imaging.. J. Cardiol..

[25] Gimelli A., Liga R., Duce V., Kusch A., Clemente A., Marzullo P. (2017). Accuracy of myocardial perfusion imaging in detecting multivessel coronary artery disease: A cardiac CZT study.. J. Nucl. Cardiol..

[26] Van Tosh A., Nichols K.J. (2023). SPECT measurements of myocardial blood flow and flow reserve: From development to implementation.. J. Nucl. Cardiol..

